# Effect of the Nd content on the structural and photoluminescence properties of silicon-rich silicon dioxide thin films

**DOI:** 10.1186/1556-276X-6-161

**Published:** 2011-02-21

**Authors:** Olivier Debieu, Julien Cardin, Xavier Portier, Fabrice Gourbilleau

**Affiliations:** 1 CIMAP, UMR CNRS/CEA/ENSICAEN/UCBN, Ensicaen 6 Bd Maréchal Juin, 14050 Caen Cedex 4, France

## Abstract

In this article, the microstructure and photoluminescence (PL) properties of Nd-doped silicon-rich silicon oxide (SRSO) are reported as a function of the annealing temperature and the Nd concentration. The thin films, which were grown on Si substrates by reactive magnetron co-sputtering, contain the same Si excess as determined by Rutherford backscattering spectrometry. Fourier transform infrared (FTIR) spectra show that a phase separation occurs during the annealing because of the condensation of the Si excess resulting in the formation of silicon nanoparticles (Si-np) as detected by high-resolution transmission electron microscopy and X-ray diffraction (XRD) measurements. Under non-resonant excitation at 488 nm, our Nd-doped SRSO films simultaneously exhibited PL from Si-np and Nd^3+ ^demonstrating the efficient energy transfer between Si-np and Nd^3+ ^and the sensitizing effect of Si-np. Upon increasing the Nd concentration from 0.08 to 4.9 at.%, our samples revealed a progressive quenching of the Nd^3+ ^PL which can be correlated with the concomitant increase of disorder within the host matrix as shown by FTIR experiments. Moreover, the presence of Nd-oxide nanocrystals in the highest Nd-doped sample was established by XRD. It is, therefore, suggested that the Nd clustering, as well as disorder, are responsible for the concentration quenching of the PL of Nd^3+^.

## Introduction

Over the last decade, there has been an increasing interest toward nanomaterials for novel applications. One of the challenging fields concerns silicon-compatible light sources which are getting more and more attractive since they can be integrated to microelectronics devices [1]. Amorphous SiO_2 _is an inefficient host matrix for the photoluminescence (PL) of Nd^3+ ^ions since, on the one hand, the absorption cross section of Nd is low (1 × 10^-20 ^cm^2^) and, on the other hand, the Nd solubility in silica is limited by clustering [2 ,3], which quenches the PL of the rare earth (RE) ions [4,5]. However, since the discovery of the sensitizing effect of silicon nanoparticles (Si-np) toward the RE ions [6], RE-doped a-SiO_2 _films containing Si-np are promising candidates for the achievement of future photonic devices. In such nanocomposites, Nd^3+ ^ions benefit from the high absorption cross section of Si-np (1-100 × 10^-17 ^cm^2^) by an efficient energy transfer mechanism, which enables the PL efficiency of RE ions to be enhanced by 3-4 orders of magnitude offering interesting opportunities for the achievement of future practical devices optically excited. In contrast to Er^3+ ^ions [6-8], such materials doped with Nd have not been widely investigated and, accordingly, the energy transfer mechanism between Si-np and Nd^3+ ^ions, and its limitation [9-16]. Several authors have demonstrated that the energy transfer is more effective with small Si-np [10,11]. Seo et al. [11] have observed a decrease of the PL intensity of Nd^3+ ^ions upon increasing the Si excess, i.e., increasing the Si-np average size. They concluded that only small Si-np which present excitonic states with a sufficient energy band-gap can excite the ^4^*F*_3/2 _level of Nd^3+ ^ions. Several groups, which studied the effect of the Nd concentration in the PL properties of Nd-doped Si-np/SiO_2 _demonstrated that the PL of Nd^3+ ^ions is more efficient at low Nd concentration [12,13].

The object of the present investigation is therefore to characterize the PL properties of nanostructured thin films containing a low concentration of Si excess as a function of the Nd concentration and the annealing temperature in relation with their microstructures. The Nd-doped silicon-rich silicon oxide (SRSO) thin layers were synthesized by reactive magnetron co-sputtering. Their microstructures were examined using high-resolution transmission electron microscopy (HRTEM), X-ray diffraction (XRD), and Fourier transform infrared (FTIR) spectroscopy. We could notably establish the proper conditions to obtain efficient PL of Nd^3+ ^but also describe its limitations.

## Experiment

In this study, Nd-doped SRSO thin layers were deposited at room temperature on *p*-type Si wafers by a reactive magnetron RF co-sputtering method that consists in sputtering simultaneously a pure SiO_2 _target topped with Nd_2_O_3 _chips. The Nd content was monitored by the surface ratio between the Nd_2_O_3 _chips and the SiO_2 _target. The sputtering gas was a mixture of argon and hydrogen; the latter enables us to control the Si excess of the deposited layers by reacting with oxide species in the plasma [17]. The samples were subsequently annealed at high temperature ranging from 900 to 1100 °C in a dry nitrogen flow.

The composition of the deposited layers was determined by Rutherford backscattering spectrometry, while microstructural analyses were performed using of XRD and HRTEM on samples prepared in the cross-sectional configuration using a JEOL 2010F (200 kV). The infrared absorption properties were investigated unsing a Nicolet Nexus FTIR spectrometer at Brewster's incidence.

Room temperature PL measurements were performed using an argon ion laser operating at 488 nm (7.6 W/cm^2^) as excitation source. This excitation wavelength is non-resonant with Nd^3+ ^ions so that only an indirect excitation of Nd can occur [13,15]. The visible spectra were recorded using a fast photomultiplier (Hamamatsu) after dispersion of the PL with a Jobin-Yvon TRIAX 180 monochromator, while the infrared PL was measured using a Jobin-Yvon THR 1000 monochromator mounted with a cooled Ge detector and a lock-in amplifier to record the near-infrared spectra up to 1.5 μm.

## Results

In this study, we were interested in four Nd-doped SRSO thin films containing the same excess of Si (7 at.%) with various Nd contents ranging from 0.08 to 4.9 at.%.

### Microstructure

**Figure 1:**
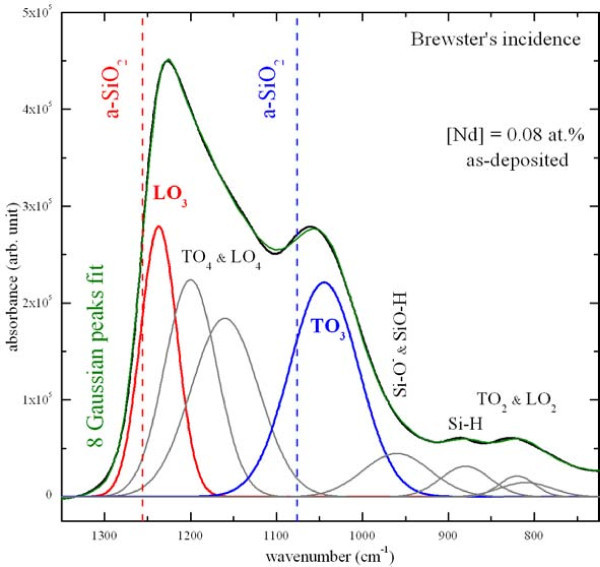
**FTIR spectrum of the lowest Nd-doped sample as-deposited**.

Figure 1 shows the FTIR spectrum of the lowest Nd-doped sample as-deposited and a fit with eight Gaussian peaks. Several bands characteristic of amorphous SiO_2 _are observed. The two prominent bands at 1236 (red), and 1052 cm^-1 ^(blue) are assigned to longitudinal optical (LO_3_) and transverse optical (TO_3_) phonons of Si-O bonds, respectively. One can notice that these two bands are slightly shifted to lower wavenumbers compared to the stoichiometric positions of a-SiO_2 _at 1256 and 1076 cm^-1^, respectively. The TO_2_, LO_2_, LO_4_, and TO_4 _vibration modes are also present at 810, 820, 1160, and 1200 cm^-1^, respectively. In addition to Si-O vibration modes, a weak absorption band centered at 880 cm^-1 ^is observed. This peak, which is assigned to Si-H bonds, disappears after annealing because of the hydrogen desorption.

**Figure 2:**
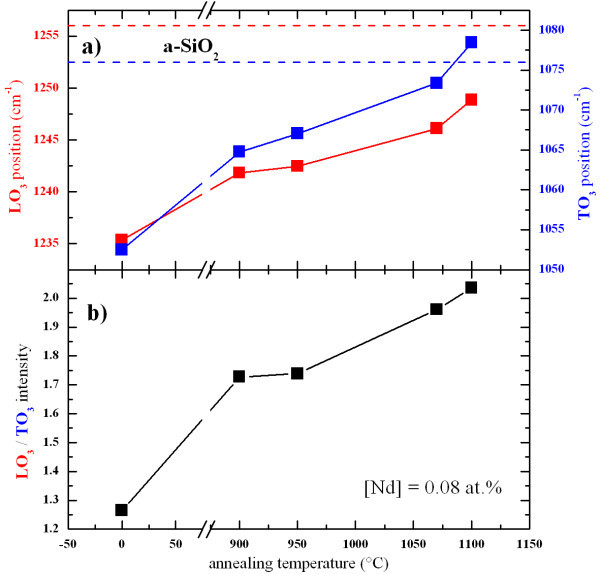
**Evolutions of the positions of the LO_3 _and TO_3 _peaks, and the LO_3_/TO_3 _intensity ratio, as a function of the annealing temperature**.

Figure 2a shows the evolution of the positions of the LO_3 _and TO_3 _vibration modes, and the LO_3_/TO_3 _intensity ratio, as a function of the annealing temperature. One can observe that, while the annealing temperature was increased, the TO_3 _and LO_3 _peaks' positions progressively shifted to higher wavenumbers toward their respective stoichiometric positions. It is explained by the phase separation that results in the formation of Si-np [18,19]. The increase of the LO_3 _band intensity (see Figure 2b) is related to the increase of the number of Si-O-Si bonds at the SiO_*x*_/Si-np interface [19,20], i.e., the increase of the density of Si-np [21].

**Figure 3:**
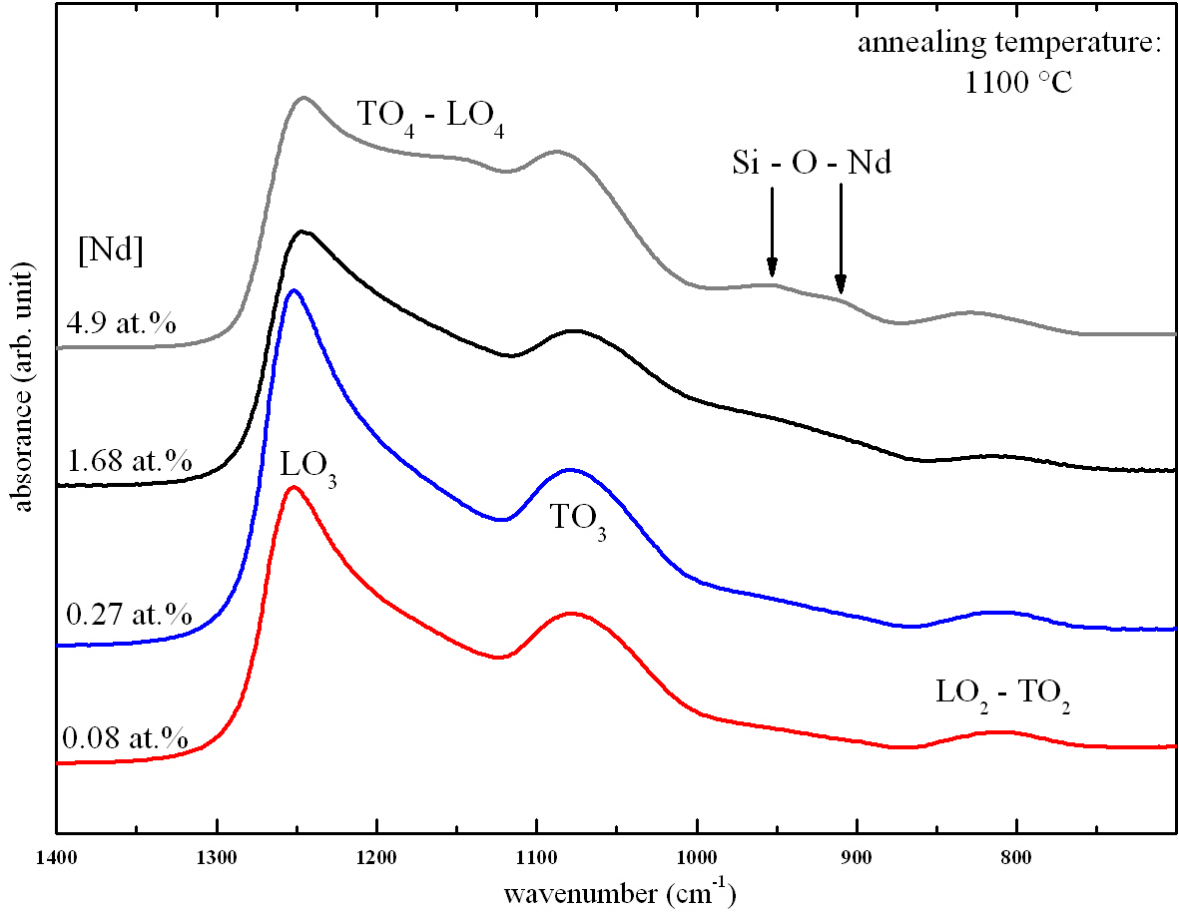
**Evolution of the FTIR spectra as a function of the Nd concentration**.

Figure 3 presents the evolution of the FTIR spectra of samples annealed at 1100 °C as a function of the Nd concentration. One can observe that the LO_3 _band intensity, which is constant at low Nd concentrations of 0.08 and 0.27 at.%, significantly decreased while the Nd content was increased from 1.68 to 4.9 at.%. This evolution contrasts with the one of the TO_4_-LO_4 _pair modes. Indeed, the TO_4_-LO_4 _intensity remains constant at low Nd concentrations of 0.08 and 0.27 at.%, and then, it progressively increases with increasing Nd content. This demonstrates that the incorporation of Nd in the thin films generates disorder in the host SiO_2 _matrix.

Moreover, one can notice, in the spectrum of the highest Nd-doped sample, the emergence of two weak absorption peaks centered at 910 and 950 cm^-1 ^which are assigned to asymmetric mode of Si-O-Nd bonds [22]. These peaks are located above a shoulder which can originate from Si-O^- ^and Si-OH phonons [23,24]. However, one can exclude the existence of the Si-OH vibration mode after annealing because of the hydrogen desorption. The emergence of these two absorption peaks suggests that other phonons are also optically active in this spectral range.

**Figure 4:**
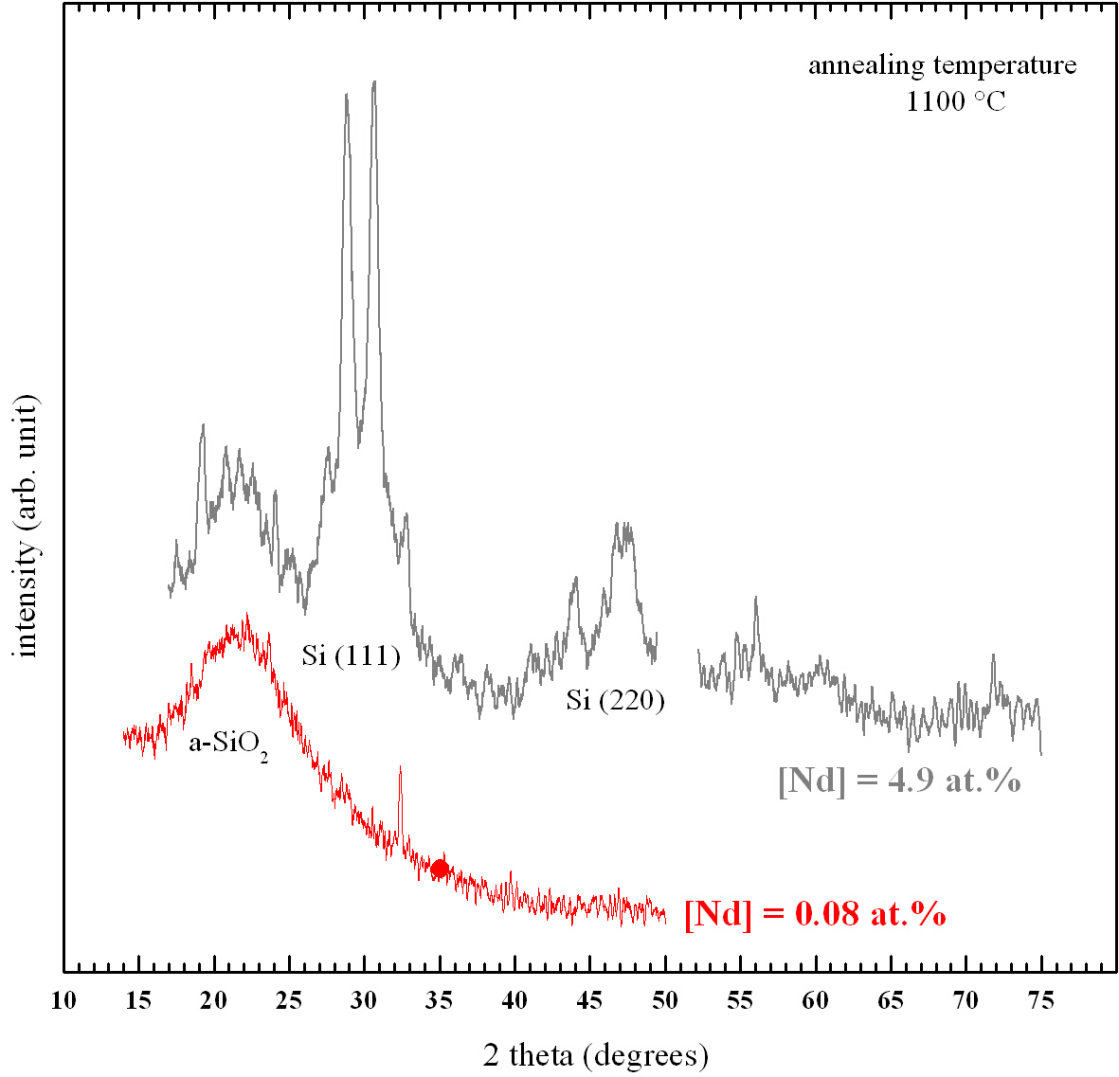
**XRD patterns of the highest and lowest Nd-doped samples annealed at 1100 °C**.

In Figure 4 is depicted the XRD spectra of the lowest and highest Nd-doped samples. In the former sample, one broad band corresponding to a-SiO_2 _is observed, while the pattern of the latter sample indicates the presence of additional phases. In the 27-32° range, it shows various sharp peaks that are located above a broad band centered at 29°. This peak, and the 48° one, indicate the presence of nanocrystalline Si [21,25], while the sharp and intense peaks located at 27.6°, 28.8°, and 30.7° are assigned to Nd_2_O_3 _crystals. However, the 28.8° peak may result from both crystalline Si and Nd_2_O_3_. It is interesting to note that the 27.6° and 30.7° peaks fairly concur with the ones observed in neodymia-silica composites containing Nd_2_O_3 _nanocrystals by several groups [2,3]. As a consequence, the presence of Nd_2_O_3 _and Si nanocrystals in the highest Nd-doped sample is established, while no crystalline phases are detected in the low Nd-doped one.

**Figure 5:**
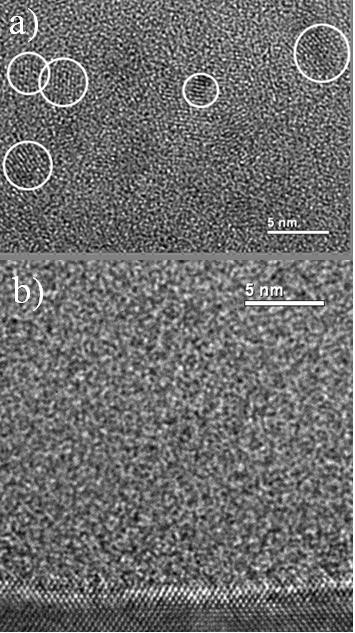
**HRTEM images of the highest (a) and lowest (b) Nd-doped samples annealed at 1100 °C**.

Figure 5 shows the HRTEM images of the two latter samples investigated by XRD after annealing at 1100 °C. In the image of the sample with the highest Nd concentration of 4.9 at.% (Figure 5a), one can recognize small Si nanocrystals because of the lattice fringes corresponding to the Si crystalline feature, while no crystalline structure was observed in the images of the film containing the lowest Nd concentration of 0.08 at.% (Figure 5b). These two images are in accordance with the XRD results (see Figure 4). However, one cannot exclude that the lowest Nd-doped sample could small contain amorphous Si-np.

### PL spectroscopy

**Figure 6:**
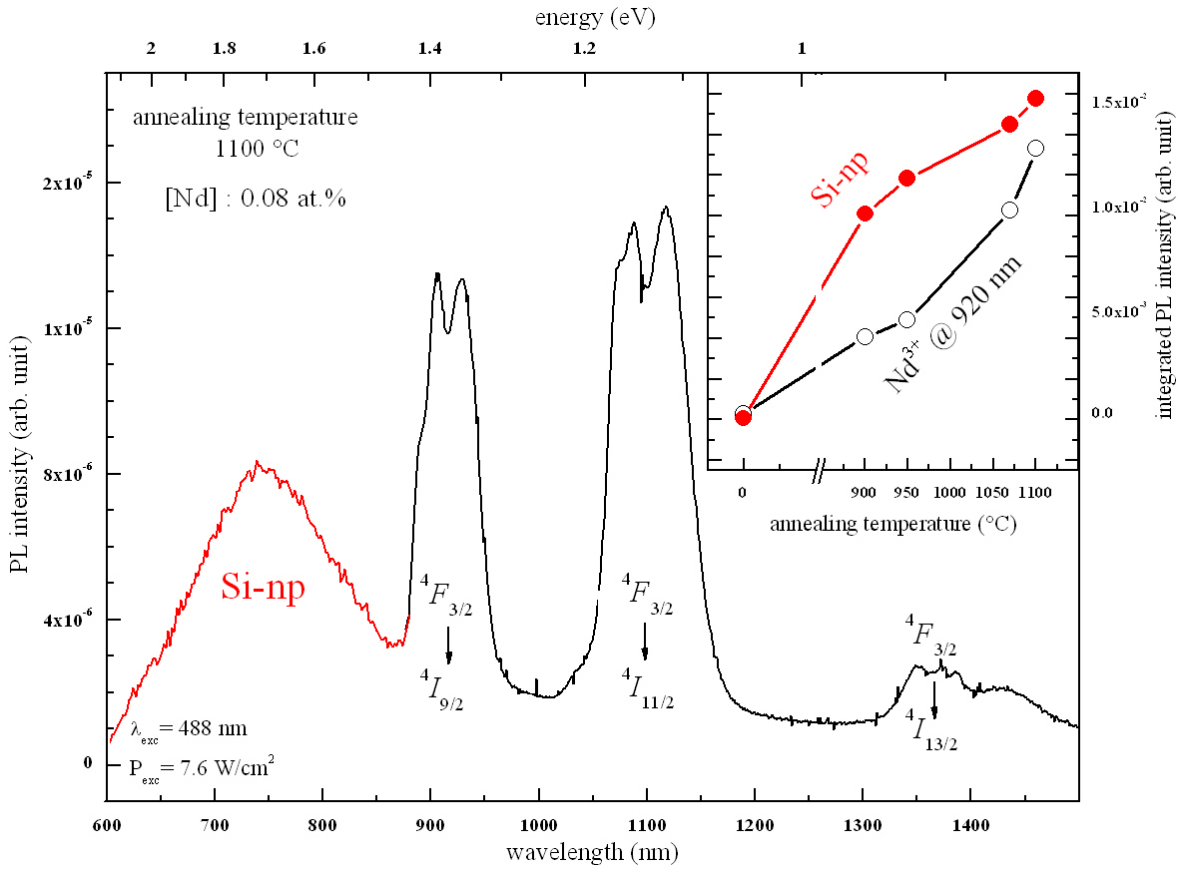
**PL spectrum of the lowest Nd-doped sample annealed at 1100 °C**. (Inset) Evolutions of the integrated PL intensity of the Si-np PL band and the first Nd^3+ ^ions PL peak as a function of the annealing temperature.

Figure 6 shows the PL spectrum of the lowest Nd-doped sample after annealing at 1100 °C. In the visible domain, one can observe a broad PL band that is originating from quantum-confined excitonic states in small Si-np, while in the infrared domain, three peaks centered at around 920, 1100, and 1350 nm are distinguishable and are attributed to the infra-4*f *shell transitions of Nd^3+ ^ions from the ^4^*F*_3/2 _level to the ^4^*I*_9/2_, ^4^*I*_11/2_, and ^4^*I*_13/2 _levels, respectively. The presence of the PL of Nd^3+ ^ions after non-resonant excitation brings to light the sensitizing effect of Si-np towards Nd^3+ ^ions.

The evolution of the integrated PL intensity of the Si-np PL band and the 920-nm PL peak is shown in the inset of Figure 6. The enhancement of the PL intensity of the broad visible PL band with the annealing temperature is characteristic for Si-np embedded in SiO_2_. It is due to the increase of the Si-np density, as shown by the increase of the LO_3 _band intensity in the FTIR spectra (see Figure 2) [21], as well as the improvement of their passivation [26] and the decrease of disorder in the host matrix. The latter is a source of non-radiative recombination channels. Interestingly, one can observe that the evolution of the PL intensity of Nd^3+ ^ions as a function of the annealing temperature is manifestly correlated with the one of Si-np. Reminding that the PL measurements were done under non-resonant excitation, this behavior underlines the strong coupling between Si-np and Nd^3+ ^ions, and, accordingly, the potential of sensitizing of Si-np. The increase of the PL intensity of Nd^3+ ^is then explained by the increase of the Si-np density as well as the increase of non-radiative de-excitation channels of both Si-np and Nd^3+^. The Nd^3+ ^PL intensity is then maximal after annealing at 1100 °C which is generally admitted as the optimal annealing temperature for the PL of Si-np.

**Figure 7:**
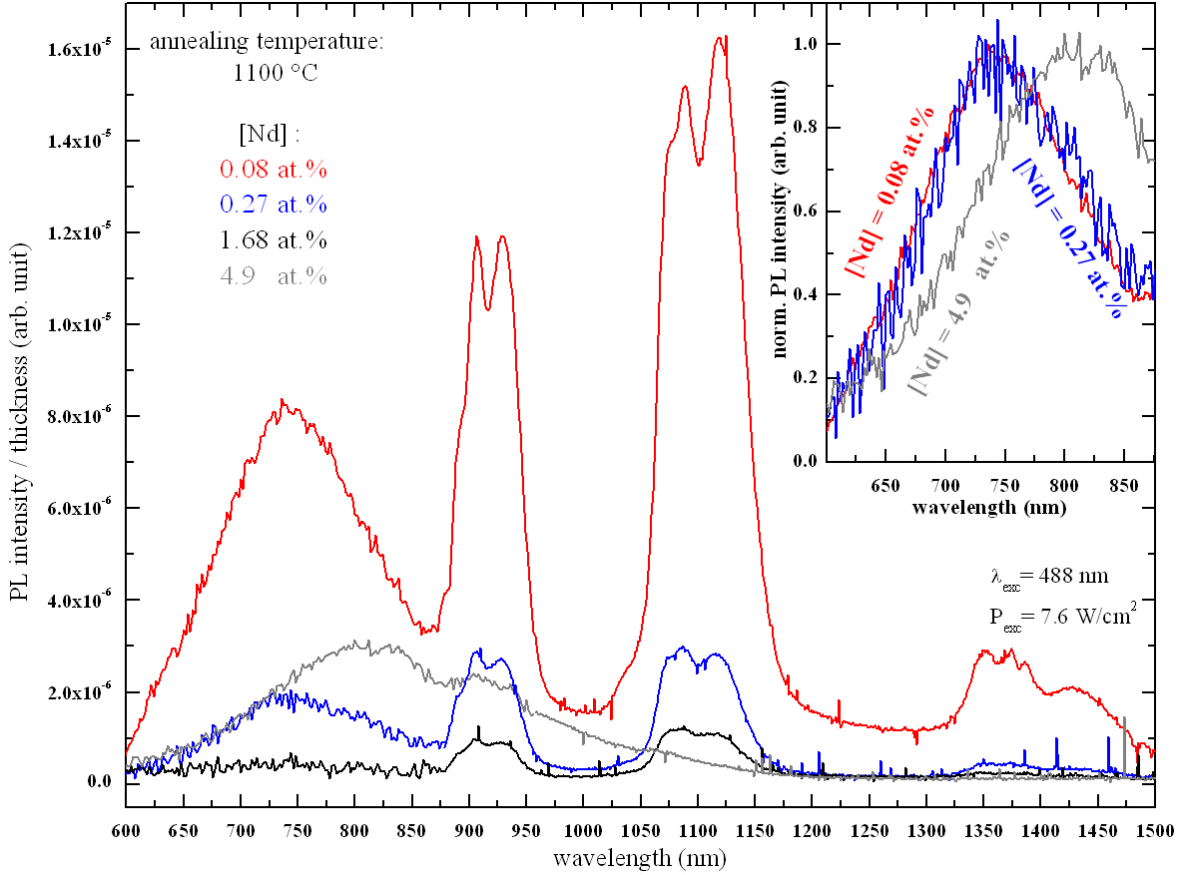
**Evolution of the PL spectra as a function of the Nd concentration**.

Figure 7 shows the behavior of the PL spectra of the thin films annealed at 1100 °C as a function of the Nd concentration. As the Nd content increases from 0.08 to 0.27 at.%, the PL intensity of Si-np drastically drops and disappears at 1.68 at.%. Then, PL of Si-np surprisingly reappears at the highest Nd concentration of 4.9 at.%. Interestingly, one can observe that the positions and widths of the PL peaks of the two lowest Nd-doped samples remain identical (see the inset); whereas the PL peak of the highest Nd-doped film is manifestly shifted to longer wavelengths. According to the quantum confinement model, the PL of the latter sample therefore emanates from Si-np that are sensibly larger than the ones present in the two former samples. In the infrared spectral domain, one can observe that the PL intensity of Nd^3+ ^ions drops progressively with increasing Nd concentration.

## Discussion

During the annealing, a phase separation occurs as demonstrated in the FTIR spectra in Figure 1, leading to the condensation of Si-np that were detected by XRD (see Figure 4) and HRTEM (see Figure 5). Besides, the presence of Si-np in the films was confirmed by the occurrence after annealing of a 740-nm broad PL band that is characteristic for Si-np.

The presence of PL of Nd^3+ ^ions under non-resonant excitation evidenced the efficient energy transfer between Si-np and Nd^3+ ^ions (Figure 6). The concentration quenching of the PL of Nd^3+ ^ions that was observed in Figure 7 is partly explained by cross relaxation processes between Nd^3+ ^ions and neighboring Nd^3+ ^ions and/or Nd_2_O_3 _nanocrystals as reported in glass matrices [4,5]. This is supported by the existence of Nd_2_O_3 _nanocrystals in the highest Nd-doped sample (see Figure 4). Besides, non-radiative channels inherent to disorder induced by the Nd incorporation (see Figure 3) can be in competition with the energy transfer mechanism between Si-np and Nd^3+ ^ions in such nanocomposite systems leading to the common decrease of the PL intensity of Nd^3+ ^and Si-np. As a consequence, the emission of Nd^3+ ^ions is more efficient while Si-np are formed, and while the Nd content is low (0.08 at.%). In such conditions, Nd^3+ ^ions benefit from the sensitizing effect of Si-np and from the weak competition of non-radiative recombinations in the host matrix. The decrease of the PL of Si-np with increasing Nd content ranging from 0.08 to 4.9 at.% (Figure 7) is explained by the raise of energy transfer between Si-np and Nd^3+ ^ions (which can be luminescent or not), and by the increase of non-radiative recombinations provided by the increase of disorder as shown in Figure 3. Besides, the presence of a Nd_2_O_3 _phase in the host matrix at the highest Nd content significantly modifies the number of oxygen atoms available to form the silicon oxide host matrix consequently leading to the formation of larger Si-np with a higher density. Besides, the formation of Nd_2_O_3 _nanocrystals results in the rise of the average interaction distance between Si-np and Nd atoms (agglomerated or not) leading to the occurrence of not-coupled Si-np, which therefore enables emission of light in the visible range. This explains the presence of the PL peak of Si-np in the highest Nd-doped sample (Figure 7) which is significantly shifted to longer wavelengths. The fact that XRD pattern of Si nanocrystals, were detected in the latter sample and not in the lowest Nd-doped sample (Figure 4) may also be attributed to the modification of the Si-np size and density.

## Conclusion

The relationships between the composition, the microstructure, and the PL properties of Nd-doped SRSO thin films that contain the same Si excess were studied. We could establish that the maximum of the PL intensity of Nd^3+ ^ions was obtained after annealing at 1100 °C which corresponds to the better situation for the achievement of highly luminescent Si-np embedded in SiO_2_, i.e., containing a small quantity of non-radiative recombination channels. It was demonstrated that the PL of Nd^3+ ^ions was quenched at high Nd-concentration (4.9 at.%) because of the formation of Nd_2_O_3 _nanocrystals and the occurrence of disorder in the host matrix. The former participates in the concentration quenching mechanism because of cross relaxation processes, while the latter induces the occurrence of new non-radiative channels which are in competition with the energy transfer mechanism between Si-np and Nd^3+ ^ions.

## Abbreviations

FTIR: Fourier transform infrared; LO: longitudinal optical; PL: photoluminescence; RE: rare earth; Si-np: silicon nanoparticles; SRSO: silicon-rich silicon oxide; TO: transverse optical; XRD: X-ray diffraction.

## Competing interests

The authors declare that they have no competing interests.

## Authors' contributions

OD fabricated the thin films and carried out the optical and microstructural characterizations. XP investigated the films by HRTEM. JC made significant contribution to the optical properties. FG conceived of the study and participated in the coordination and writing of the manuscript. All authors read and approved the final manuscript.
